# Paeonol Oxime Inhibits bFGF-Induced Angiogenesis and Reduces VEGF Levels in Fibrosarcoma Cells

**DOI:** 10.1371/journal.pone.0012358

**Published:** 2010-08-23

**Authors:** Hyo-Jeong Lee, Seung-Ae Kim, Hyo-Jung Lee, Soo-Jin Jeong, Ihn Han, Ji Hoon Jung, Eun-Ok Lee, Shudong Zhu, Chang-Yan Chen, Sung-Hoon Kim

**Affiliations:** 1 College of Oriental Medicine, Kyung Hee University, Seoul, Republic of Korea; 2 Beth Israel Deaconess Medical Center, Harvard Medical School, Boston, Massachusetts, United States of America; Yale Medical School, United States of America

## Abstract

**Background:**

We previously reported the anti-angiogenic activity of paeonol isolated from Moutan Cortex. In the present study, we investigated the negative effect of paeonol oxime (PO, a paeonol derivative) on basic fibroblast growth factor (bFGF)-mediated angiogenesis in human umbilical vein endothelial cells (HUVECs) (including tumor angiogenesis) and pro-survival activity in HT-1080 fibrosarcoma cell line.

**Methodology/Principal Findings:**

We showed that PO (IC_50_  = 17.3 µg/ml) significantly inhibited bFGF-induced cell proliferation, which was achieved with higher concentrations of paeonol (IC_50_ over 200 µg). The treatment with PO blocked bFGF-stimulated migration and *in vitro* capillary differentiation (tube formation) in a dose-dependent manner. Furthermore, PO was able to disrupt neovascularization *in vivo*. Interestingly, PO (25 µg/ml) decreased the cell viability of HT-1080 fibrosarcoma cells but not that of HUVECs. The treatment with PO at 12.5 µg/ml reduced the levels of phosphorylated AKT and VEGF expression (intracellular and extracelluar) in HT-1080 cells. Consistently, immunefluorescence imaging analysis revealed that PO treatment attenuated AKT phosphorylation in HT-1080 cells.

**Conclusions/Significance:**

Taken together, these results suggest that PO inhibits bFGF-induced angiogenesis in HUVECs and decreased the levels of PI3K, phospho-AKT and VEGF in HT-1080 cells.

## Introduction

Angiogenesis in embryonic development, reproduction and wound healing is tightly regulated by the balance between the angiogenic inhibitors and activators [1], [2], [3], [4]. The obligatory dependence of solid tumor growth and progression on angiogenesis are well supported and accepted [5]. Basic fibroblast growth factor (bFGF) and vascular endothelial growth factor (VEGF) have been well documented as angiogenic activators [6], [7], [8]. While VEGF is a primary mediator of angiogenic responses, bFGF is also one of potent angiogenesis inducers to stimulate the vascular endothelial mitogenesis and is often involved in pathologic angiogenesis. bFGF is routinely employed as angiogenic polypeptide for experimental studies [9].

Tumor angiogenesis involves complex and multiple vascular endothelial responses as well as contributions from tumor cells. The endothelial responses include angiogenic factor-stimulated changes in the vascular endothelial permeability, degradation of the basement membrane by metalloproteases, migration, remodeling and proliferation of endothelial cells to form capillary tubes, and other processes. On the other hand, tumor cells or host stromal cells express VEGF induced by hypoxia and other autocrine or paracrine growth factors [2], [3], [4], [6], [7], [8], [9] to stimulate endothelial progenitor cells for angiogenesis. Interruptions of one or more steps in these processes can negatively affect angiogenesis and inhibit early malignant lesions to lower the cancer risk [10] VEGF expression is mediated by the phosphoinositide 3-kinase (PI3K) pathway in endothelial cells [11]. Also, PI3K pathway reveals its effects through its downstream kinase AKT to regulate various cellular processes including angiogenesis [12].

**Figure 1:**
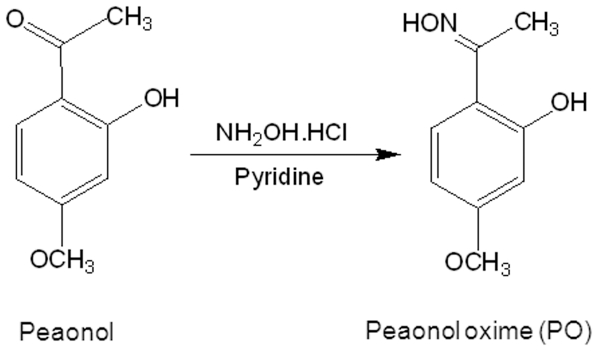
Chemical structure of paeonol oxime. Paeonol oxime (PO) (1-[2-hydroxy-4-methoxy phenyl] ethanone oxime) was synthesized from paeonol.

Paeonol oxime (1-[2-hydroxy-4-methoxy phenyl] ethanone oxime; PO) was derived from paeonol (2-hydroxy, 4-methoxy acetophenone), which has been shown to be anti-angiogenic [13] (Fig. 1). We designed and synthesized PO so that it also shares some similarity with acetylsalicylic acid (Fig. 1), a well known anti-inflammatory pain killer but also with anti-angiogenic activity [1], [14], with the goal to achieve better solubility and anti-angiogenic potency than paeonol. In this regard, we tested the anti-angiogenic properties of PO on bFGF-stimulated human umbilical vein endothelial cells (HUVECs) by examining proliferation, migration, tube formation and chick chorioallantoic membrane (CAM). We also explored the potential signaling events affected by PO in angiogenic HT-1080 cells by Western blotting and immunofluorescence microscopy.

## Results

### PO inhibited bFGF-induced proliferation of HUVECs

**Figure 2:**
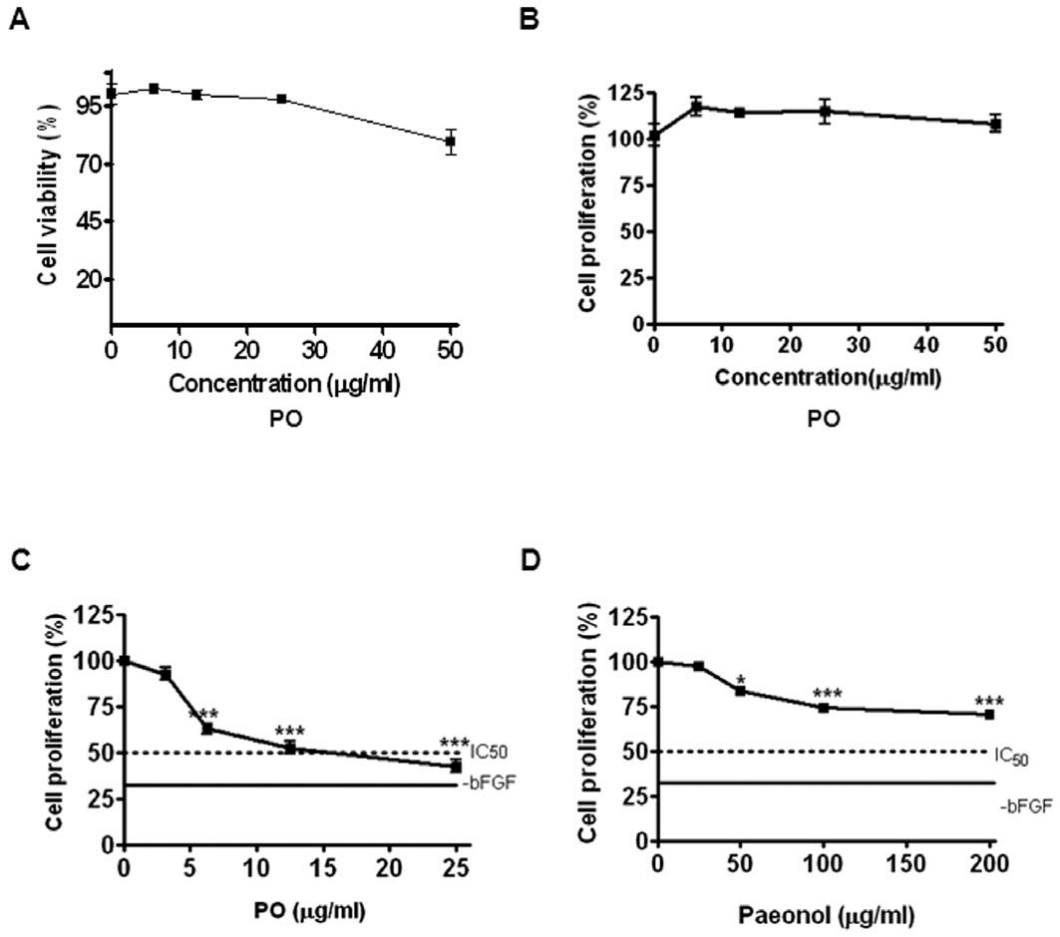
PO inhibits bFGF-induced proliferation of HUVECs. (A) Cytotoxic effect of PO in HUVECs. Cells (5×10^3^ cells/well) were seeded onto 0.1% gelatin-coated 96-well plates, starved for 6 h in M199 containing 5% heat-inactivated FBS and treated with various concentrations of PO (0, 6.125, 12.5, 25 or 50 µg/ml) for 24 h. Cell viability was measured by MTT assay. (B) Effect of PO on the proliferation of HUVECs. Cells (3×10^3^ cells/well) were treated with various concentrations of PO (0, 6.125, 12.5, 25 or 50 µg/ml) for 48 h. (C) Effect of PO on the proliferation of bFGF treated HUVECs. Cells (3×10^3^ cells/well) were treated with various concentrations of PO (0, 6.125, 12.5, 25 or 50 µg/ml) in the absence or presence of bFGF (10 ng/ml) for 48 h. (D) Effect of paeonol on the proliferation of bFGF treated HUVECs. Cells (3×10^3^ cells/well) were treated with various concentrations of paeonol (0, 25, 50, 100 or 200 µg/ml) in the absence or presence of bFGF (10 ng/ml) for 48 h. Cell proliferation assay was performed using a 5-bromo-2′-deoxyuridine (BrdU) colorimetric assay kit. The statistically significant differences between control and PO treated groups were calculated by the Student's *t-*test. ^*^, p<0.05 and ^***^, p<0.001 *versus* control.

The cytotoxicity of PO against non-proliferating HUVECs (absence of angiogenic factors) was first tested to define the range of non-toxic concentrations of PO for *in vitro* angiogenesis experiments. As shown in Fig. 2A, after 24 h incubation, PO decreased MTT metabolizing ability (metabolic viability) of HUVECs in a concentration-dependent manner, with less than 20% decrease at up to 50 µg/ml of PO. All subsequent angiogenesis experiments were carried out at even lower concentrations (6.25, 12.5 or 25 µg/ml) to make sure of absence of significant cell death in the assays. Consistent with the results of cytotoxicity assay, 5-bromo-2′-deoxyuridine (BrdU) proliferation assay further confirmed non-toxic effect of PO in HUVECs (Fig. 2B).

Since angiogenic factor-stimulated endothelial cell proliferation is a key step of the angiogenic processes [7], the effect of PO on bFGF (10 ng/ml) induced mitogenic response in HUVECs at non-toxic concentrations was analyzed. PO significantly inhibited bFGF-induced proliferation of HUVECs with IC_50_ of 17.3 µg/ml (Fig. 2C). In comparison, the parental compound paeonol suppressed bFGF-induced proliferation of HUVECs with an IC_50_ expected to be over 200 µg/ml (Fig. 2D). The marked difference observed here between PO and paeonol rendered further evaluation of the impact of PO on more of the endothelial responses induced by bFGF.

### PO inhibited bFGF-induced migration and tube formation of HUVECs

**Figure 3:**
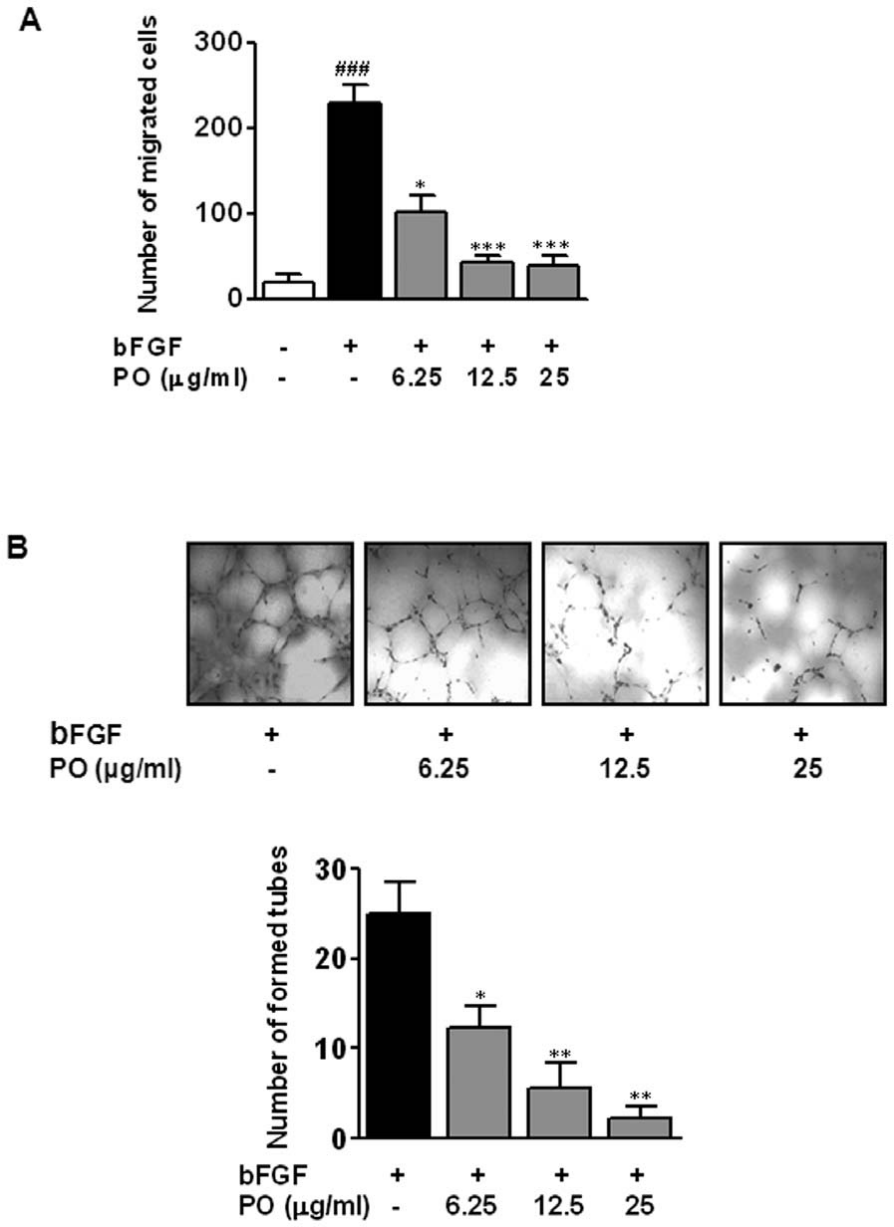
PO inhibits bFGF-induced migration and tube formation of HUVECs. Cell migration through gelatin-coated filters was measured by using the Boyden chamber. HUVECs (5×10^4^ cells/well) were plated into the upper chamber with or without various concentrations of PO and incubated for 6 h at 37°C in a 5% CO_2_ incubator. Cells migrated to the lower surface were photographed randomly under an Axiovert S 100 light microscope at ×100 magnification and counted. (B) Tube formation assay was performed using growth factor reduced Matrigel. Cells were fixed with Diff-Quick solution, photographed randomly under an Axiovert S 100 light microscope at ×100 magnification and counted. All data were expressed as mean ± S.D. The statistically significant differences between control and PO treated groups were calculated by the Student's *t-*test. ^###^, p<0.001 *versus* untreated control.^*^, p<0.05 and ^**^, p<0.01 *versus* bFGF control.

To explore the effects of PO on bFGF-stimulated angiogenesis, the migration assay using the Boyden chamber was performed. As shown in Fig. 3A, cell motility of HUVECs was increased by 10.9-fold after bFGF treatment as compared with untreated control. In contrast, PO significantly inhibited bFGF-induced migration of HUVECs, in a concentration-dependent manner with IC_50_ of 5.3 µg/ml.

It is known that HUVECs undergo extensive *in vitro* differentiation to form capillary-like tubes on the Matrigel and bFGF stimulates this process [5]. In our experiments, PO significantly inhibited bFGF-induced tube formation by HUVECs, with IC_50_ of 6.2 µg/ml (Fig. 3B). Together, these data demonstrated that PO was able to inhibit critical processes involved in angiogenic responses.

### PO disrupted bFGF-induced angiogenesis in CAM assay

**Figure 4:**
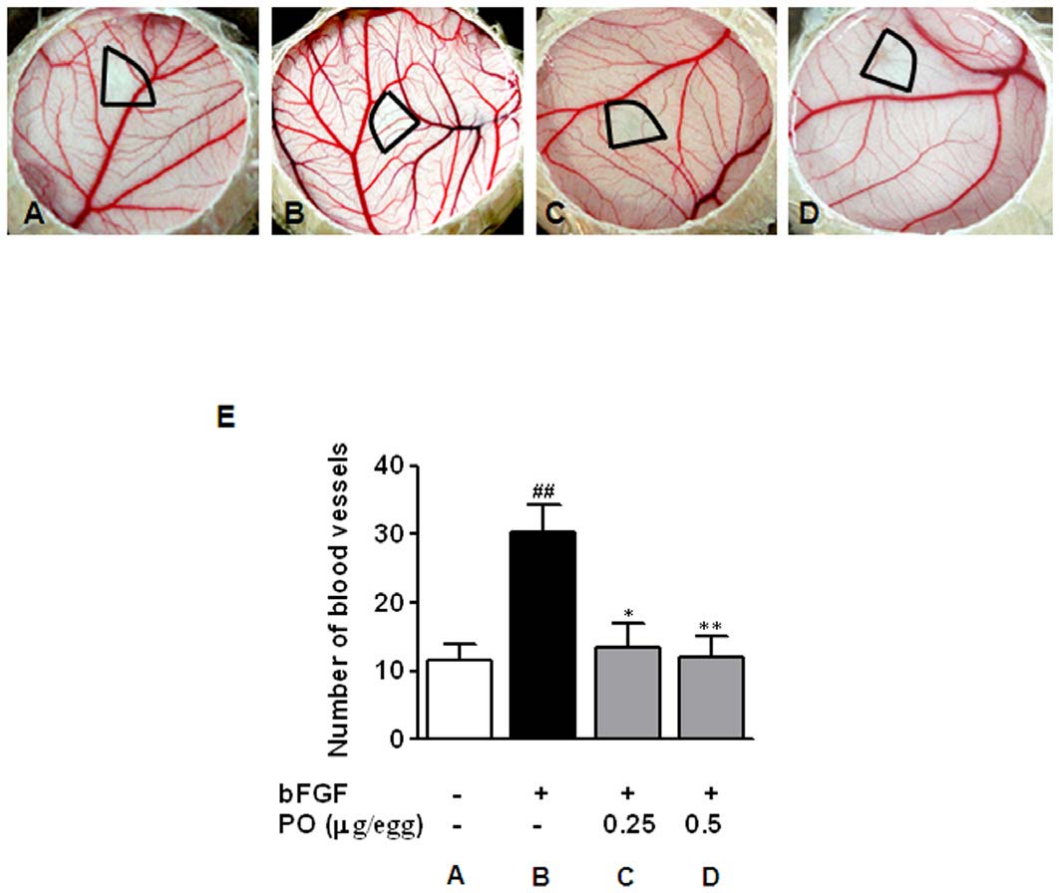
PO disrupts bFGF-induced angiogenesis *in vivo*. PO and bFGF were loaded on CAMs of 10-day-old fertilized chicken eggs. After 72 h incubation, a fat emulsion was injected into the CAMs for better visualization of the blood vessels. Thermanox disc and surrounding CAMs were photographed. (A) Untreated control, (B) bFGF-treated control, (C) 0.25 µg of PO with bFGF, and (D) 0.5 µg of PO with bFGF. (E) The number of newly formed blood vessels per field was counted. All data are presented as mean ± S.D., *n* = 5. Statistically significant differences between control and sample groups were calculated by Student's *t*-test. ^##^, p<0.05 *versus* untreated control.^*^, p<0.05 and ^***^, p<0.001 *versus* bFGF control.

To confirm the anti-angiogenic potential of PO, chick CAM assay was employed in the physiological context of vascular endothelial cells within intact vessels. As shown in Fig. 4, the treatment with bFGF (100 ng/embryo) resulted in a 2.6-fold increase of new blood vessels under the thermonox disc applied on the CAMs. Furthermore, PO treatment at both doses of 0.25 and 0.5 µg/egg almost totally nullified bFGF-induced angiogenesis without apparent thrombosis and hemorrhage.

### PO downregulated the expression of VEGF and PI3K/AKT signaling in HT-1080 cells

**Figure 5:**
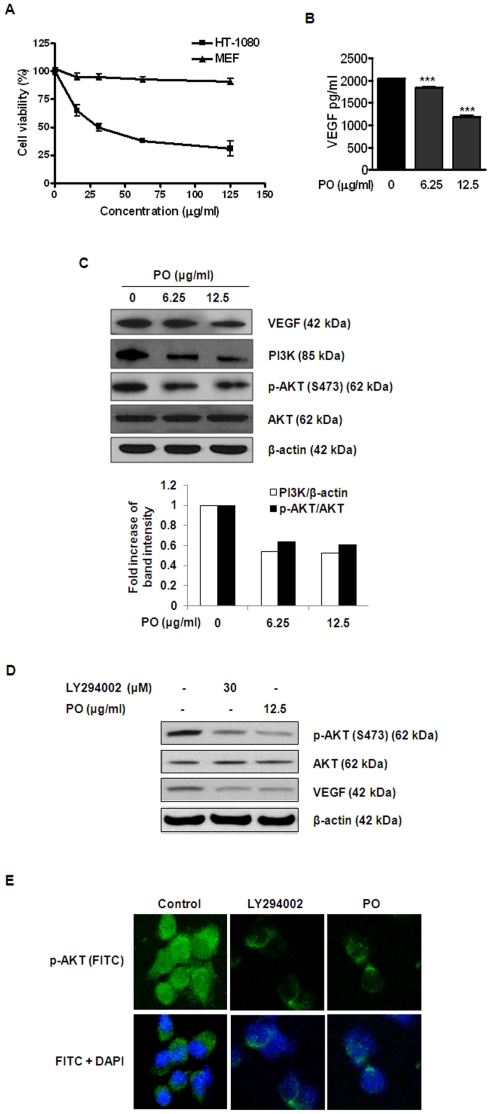
PO downregulates the expression of VEGF and the PI3K/AKT signaling in HT-1080 cells. (A) HT-1080 cells were treated with PO for 24 h and the cell viability was assessed by MTT assay. (B) The conditioned medium from HT-1080 cells treated with or without PO for 24 h was used for VEGF measurement using an ELISA kit. Level of VEGF was normalized to the cell number of each sample after 24 h of PO treatment. ^***^, p<0.001 *versus* control. (C) HT-1080 cells were treated with PO (0, 6.25 or 12.5 µg/ml) for 24 h. The levels of VEGF, PI3K, phospho-AKT and AKT were determined by Western blotting. (D) HT-1080 cells were treated with LY294002 or PO for 24 h and subjected to Western blotting for VEGF, phospho-AKT and AKT. (E) Cells were treated with LY294002 or PO as described in (D) and incubated with anti-phospho-AKT and FITC conjugated secondary antibodies. The immunostained cells were mounted in medium containing DAPI and visualized under a Carl Zeiss LSM5 confocal microscope.

The effect of PO on human HT-1080 fibrosarcoma cells (a highly angiogenic cancer cell line) was investigated. Interestingly, PO, at 20 µg/ml, induced apparent cytotoxicity in the HT-1080 fibrosarcoma cells but not in normal mouse embryonic fibroblasts (MEF) (Fig. 5A). This decrease in cell viability upon PO treatment was also concentration dependent (Fig. 5A). Since 20 µg/ml PO did not induce apparent cytotoxicity in non-proliferating HUVECs either (Fig. 2A), the result suggests that besides roles in angiogenesis, PO at certain concentration might selectively affect viability of certain tumor cells.

Since VEGF is a crucial mediator of angiogenesis, we examined whether PO affects the expression of VEGF in HT-1080 cells. PO at 12.5 µg/ml significantly reduced not only the level of the secretion (Fig. 5B) its but also the expression of VEGF (Fig. 5C).

PI3K/AKT pathway is known to play a crucial role in angiogenic responses [15], [16]. Thus, we examined the potential role of PO on activation of PI3K and AKT in HT-1080 cells. As shown in Fig. 5C, PO at 12.5 µg/ml led to the decreased expression of PI3K and phosphorylation of its downstream target AKT. In order to confirm the ability of PO to inhibit PI3K/AKT pathway, a PI3K inhibitor LY294002 was also used as a positive control for inhibiting PI3K/AKT signaling in HT-1080 cells. As expected, LY294002 significantly suppressed AKT phosphorylation and VEGF expression similar to PO treatment (Fig. 5D). This was further verified by the immunofluorescence microscopy of HT-1080 cells in presence and absence of LY294002 and PO treatment (Fig. 5E).

**Figure 6:**
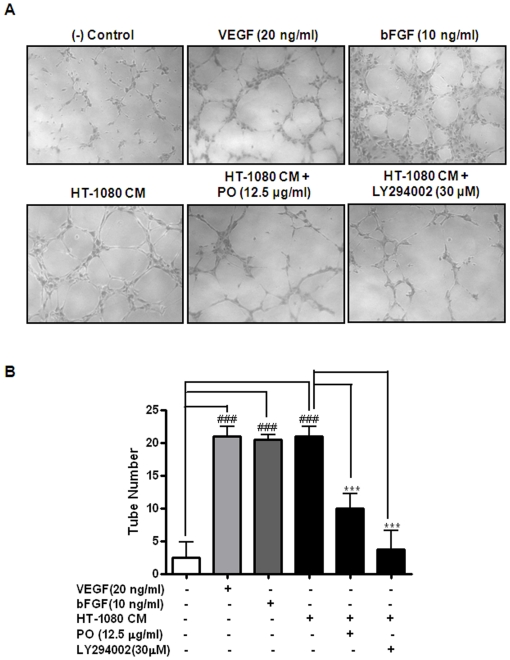
PO inhibits the tube formation of HUVECs induced by culture supernatants of HT-1080 cells. Tube formation assay was performed using growth factor reduced Matrigel. (A) Cells were fixed with Diff-Quick solution and photographed randomly under an Axiovert S 100 light microscope at ×100 magnification. (B) Tube networks were quantified using NIH Scion image program. All data were expressed as mean ± S.D. The statistically significant differences between control and PO treated groups were calculated by the Student's *t-*test. ^###^, p<0.001 *versus* untreated control in M199 medium. ^***^, p<0.001 *versus* culture supernatant from HT-1080.

To further confirm that the effects of PO on HT-1080 cells are due to its action on angiogenic properties, *in vitro* tube formation assay was carried out in the cells with or without the treatment with PO for 24 h (Fig. 6). The culture medium (CM) from HT-1080 cells induced HUVEC tube formation compared with that from untreated control cells (lanes 1 and 4). In contrast, less than half of PO-treated cells grown in CM (>47%) were able to induce HUVEC tube formation, than that cultured in CM alone (lanes 4 and 5), suggesting that PO is a potent anti-angiogenic agent. LY294002 was also prevented the tube formation (lane 6). VEGF and bFGF served as positive controls (lanes 2 and 3). However, PO had no significant effect on HT-1080 mediated cell proliferation and VEGF secretion in HUVECs (data not shown).

## Discussion

Paeonol was reported to have multi-biological activities such as anti-inflammatory [17], [18], anti-allergic [19], anti-tumor [20], anti-angiogenic [13] and anti-oxidant [21], [22] activities. In the present study, PO was synthesized from paeonol with an anticipation of improved solubility and increased anti-angiogenic potential based on the similarity of PO to aspirin. Assays based on HUVECs clearly demonstrated the improved anti-proliferative activity of PO compared with paeonol. Specifically, PO inhibited bFGF-induced proliferation of HUVECs with IC_50_ of 17.3 µg/ml, indicating that PO is more potent to inhibit cell proliferation than paeonol (IC_50_ >200 µg/ml). Also, PO suppressed HUVEC motility and tube formation in a concentration dependent manner and with similar potency (i.e., IC_50_ = 6.2 µg/ml). Consistently, PO significantly disrupted the neovascularization in bFGF-treated CAM of developing embryos *in vivo*. Furthermore, PO was effective at dosage of 0.25 and 0.5 µg per egg, more sensitive than paeonol, which was effective at 0.8 and 1.6 µg per egg.

Interestingly, PO displayed cytotoxicity against HT-1080 cells more effectively than HUVECs and MEFs, indicating a possible selectivity of PO to kill tumor cells over normal cells. It is possible that the cytotoxicity of PO against HT-1080 observed in Fig. 5A could also be due to/associated with the inhibition of PI3K/AKT signaling (Fig. 5D). In future, we plan to further study more of this interesting phenomenon and what it might implicate in potential clinical interventions. In terms of the molecular mechanisms responsible for anti-angiogenic response, VEGF production promotes the carcinogenic progression of cancer cells [23], [24], and VEGF is an important cytokine involved in both vasculogenesis and angiogenesis [25]. We observed that PO inhibited extracellular production and intracellular expression of VEGF in HT-1080 cells. These suggest PO would be able to suppress angiogenic processes in HT-1080 cancer cells as well as in human endothelial cells, especially considering that it has been reported that HT-1080 cell-secreted VEGF, promoted the basement membrane degradation, migration, proliferation and tube formation of endothelial cells [26], although we have not explored further on this due to our focus of study on HUVEC angiogenesis in this report. In other words, the downregulation of VEGF signaling by PO observed in HT-1080 cells is likely to lead to inhibition on angiogenesis of fibrosarcoma.

PI3K/AKT pathway has been known to stimulate angiogenesis [27], [28]. Arbiser et al. reported that Solenopsin, the alkaloidal component of the fire ant, prevented angiogenesis by inhibiting the activation of PI3K and the phosphorylation of AKT [29]. Nakashio et al. also reported that Topotecan inhibits VEGF- and bFGF-induced vascular endothelial cell migration via downregulation of the PI3K-AKT signaling pathway [30]. Similarly, we also showed that PO reduced the expression of PI3K, subsequently downregulated the phosphorylation of AKT, and VEGF secretion in HT-1080 sarcoma cells. A PI3K inhibitor LY294002 significantly suppressed AKT phosphorylation and VEGF expression, which is similar to PO treatment, implying that PI3K is related to and downstream of VEGF in the cells. Furthermore, the results from *in vitro* capillary tube formation assay, indicated that PO is able to inhibit HT-1080-mediated tube formation. Since LY294002 also had a similar inhibitory effect on tube formation in the same cells, it suggests that PO exerts its anti-angiogenic activity, possibly via VEGF/PI3KAKT pathway. Further studies are under way to examine whether PO-mediated anti-angiogenic activity is associated with this signaling pathway in HUVEC and cancer cells.

In summary, we demonstrated that PO inhibited bFGF-stimulated proliferation, migration, tube formation in human endothelial cells and also disrupted *in vivo* neovascularization. We also showed that PO suppressed PI3K/AKT signaling and reduced VEGF secretion and expression fibrosarcoma HT-1080 cells, which is consistent with the current understanding of the role of VEGF/PI3K in angiogenesis. The results from tube formation assay further indicate the anti-tumor angiogenic effect of PO. Based on our study, PO appears to be a promising angiogenic inhibitor and applicable to angiogenesis related diseases. Our data also suggested that PI3K is an intracellular target of PO to for achieving its inhibition on angiogenesis.

## Materials and Methods

### Paeonol oxime

Paeonol oxime (1-[2-hydroxy-4-methoxy phenyl] ethanone oxime, PO; molecular weight: 213) was synthesized from paeonol (Fig. 1A). Paeonol, hydroxyl ammonium and pyridine in anhydrous ethanol were stirred for 24 h under reflux and the reaction mixture was concentrated by Rotary evaporator. The resulting residue was purified by column chromatography. PO was more soluble as compared with paeonol due to the extra hydroxyl radical.

### Cell culture

Human HT-1080 fibrosarcoma cells (ATCC CCL-121) were purchased from ATCC (Rockville, MD). The cells were cultured in RPMI-1640 supplemented with 10% heat-inactivated fetal bovine serum (FBS) and 100 units/ml of antibiotic-antimycotics. HUVECs were isolated from fresh human umbilical cord veins according to a published protocol [31] and cultured in M199 supplemented with 20% heat-inactivated FBS, 3 ng/ml bFGF, 5 units/ml heparin and 100 units/ml antibiotic-antimycotic in 0.1% gelatin coated flasks. HUVECs were used in passages three to six.

### Cytotoxicity assay

MTT [3-(4,5dimethylthiazol-2-yl)-2,5-diphenyltetrazolium bromide] assay was performed to determine the cytotoxicity of paenol oxime against non-proliferative HUVECs, mouse embryonic fibroblast (MEF) and HT-1080 cells [2]. Briefly, cells were seeded into 96-well plates at a density of 1×10^4^ cells per well, grown in presence of various concentrations (0 ∼120 µg/ml) of PO for 24 h. MTT solution (1 mg/ml) was added to each well and incubated at 37°C for 4 h. The medium was removed, and formazan formed was solubilized with DMSO and the absorbance at 570 nm measured by a microplate reader (Molecular Devices Co., Sunnyvale, CA). The percentage of viable cells was estimated compared with untreated control.

### Proliferation assay

Cell proliferation assay was performed using a 5-bromo-2′-deoxyuridine (BrdU) colorimetric assay kit according to the manufacturer's instructions. HUVECs (3×10^3^ cells/well) were seeded into 0.1% gelatin-coated 96-well plates and incubated in a humidified incubator for 24 h. Cells were starved for 6 h in M199 containing 5% heat-inactivated FBS and then treated with various concentrations of PO or paeonol in the absence or presence of bFGF (10 ng/ml) in M199 containing 5% heat-inactivated FBS and 5 units/ml heparin at 37°C for 48 h. Cells were added with BrdU for additional 6 h, fixed with FixDenat solution for 30 min and incubated with anti-BrdU followed by the horseradish peroxidase reaction. The reaction was terminated by addition of 25 µl of 1 M H_2_SO_4_ and the absorbance was measured using a microplate reader (Molecular Devices Co., Sunnyvale, CA) at 450 nm–690 nm. IC_50_ of PO paeonol was calculated with GraphPad Prism 5.0 software.

### Migration assay

The motility of HUVECs to pass through gelatin-coated filters was measured by using the Boyden chamber (Nuero Probe, Inc., Cabin John, MD). Briefly, polyester membrane (12 µm pores) was coated with 0.1% gelatin for 30 min and dried. The lower chamber was filled with 30 µl of M199 containing 0.5% BSA in the presence or absence of bFGF (10 ng/ml). The coated membrane and upper chamber were laid over the lower chamber. HUVECs (5×10^4^ cells/well) were plated onto the upper chamber with or without various concentrations of PO and incubated for 6 h at 37°C in a 5% CO_2_ incubator. After incubation, the membrane was fixed with Diff-Quick fixative and stained with Diff-Quick Solution I and II (DADE Behring Inc., Newark, DE). The magnitude of the migrated cells was evaluated by counting the migrated cells in 2 random high-power (×100) microscope fields per well under an Axiovert S 100 light microscope (Carl Zeiss Inc., Weimar, Germany) at ×100 magnification.

### *In vitro* tube formation assay

Tube formation assay was performed using growth factor reduced Matrigel as previously described [3], [4], [32]. HUVECs (7×10^4^ cells) were seeded onto growth factor reduced Matrigel-coated 24-well plates and treated with or without various concentrations of PO in M199 with 1% FBS, 10 ng/ml bFGF and 5 units/ml heparin for 24 h at 37°C, or culture medium (CM) from HT-1080 cells treated with or without PO treatment. The cells were fixed with Diff-Quick solution and visualized under an Axiovert S 100 light microscope (Carl Zeiss Inc., Weimar, Germany) in 3 random high-power (×100) microscope fields per well. The number of circular tubules was counted by using Image-Pro® Plus 4.5 (Media CyberMetics Inc., Bethesda, MD) software.

### Chick chorioallantoic membrane (CAM) assay

*In vivo* anti-angiogenic activity of PO was assessed using CAM assay as described previously [32]. Briefly, PO and bFGF (100 ng) were loaded onto a 1/4 piece of thermonox disc (Nunc, Naperville, IL). The dried thermonox disc was applied to the CAM of a 9-day-old embryo and incubated for 48 h. Then fat emulsion was injected under the CAM for better visualization of the blood vessels. The number of newly formed blood vessels was counted. The experiment was repeated twice with 15 eggs per group.

### Enzyme linked immunosorbent assay (ELISA) for VEGF

ELISA was performed using Biosource Human Vascular Endothelial Growth Factor ELISA kit (Biosource International Inc., Camarillo, CA), following manufacturers' instructions. The minimum detectable dose of VEGF is <5 pg/ml, and 100% cross-reactivity with human VEGF-121 and complete parallelism with human VEGF-165. Percentages of coefficient of variation CV) in intra- and inter-assay were 3.7–5.5% and 6.5–9.3%, respectively. All samples were within the dynamic range of the standard curve.

### Western blot analysis

Cell lysate preparation and immunoblotting were as described previously [3], [32]. In brief, HT-1080 cells were lyzed in lysis buffer containing 50 mM Tris-HCl (pH 7.4), 150 mM NaCl, 1% Triton X-100, 0.1% SDS, 1 mM EDTA, supplemented with protease inhibitors (10 µg/ml leupeptin, 10 µg/ml aprotinin, 10 µg/ml pepstatin A, and 1 mM of 4-(2-aminoethyl) benzenesulfonyl fluoride) and phosphatase inhibitors (1 mM NaF and 1 mM Na_3_VO_4_). The extracts were incubated on ice for 20 min, centrifuged at 14000× *g* for 20 min at 4°C and supernatants were collected. Protein conntraions were determined by Bradford assay (Bio-Rad), and proteins were separated by electrophoresison SDS-polyacrylamide gels and transferred to Hybond-C nitrocellulose membranes at 300 mA for 2 h. Membranes were blocked with 5% nonfat dry milk in TBS-T (0.05% Tween-20, 138 mM NaCl, and 25 mM Tris base). Protein expression was determined using antibodies against VEGF (Santa Cruz Biotechnologies, Santa Cruz, CA), P13K, phospho-AKT, AKT (Cell Signaling Technology, Danvers, MA) and β-actin (Sigma, St. Louis, MO). The blots were incubated with horseradish peroxidase-conjugated antibodies and detected by enhanced chemiluminescence (ECL) detection system.

### Immunofluorescence microscopy

HT-1080 cells were fixed on poly-L-lysine coated slides in 4% paraformaldehyde and then permeabilized in cold methanol. Cells were then incubated with 10% normal goat serum in PBS for 1 h, followed by immunostaining with rabbit polyclonal phospho-AKT antibody (Cell Signaling Technology, Danvers, MA). Rabbit IgG fluorescein isothiocyanate (FITC) antibody H & L (Abcam, Cambridge, MA) was used as the secondary antibody. The immunostained cells were mounted in medium containing DAPI (Vectashield, Vector Labs, Burlingame, CA) and visualized under a Carl Zeiss LSM5 confocal microscope.

### Statistical analysis

All data were presented as mean ± standard deviation (S.D.). The statistically significant differences between control and PO treated groups were calculated by Student's *t-*test.
